# Unpacking pathways to diversified livelihoods from projects in Pacific Island coastal fisheries

**DOI:** 10.1007/s13280-022-01727-x

**Published:** 2022-03-22

**Authors:** Matthew B. Roscher, Hampus Eriksson, Daykin Harohau, Senoveva Mauli, Jeremie Kaltavara, Wiebren J. Boonstra, Jan van der Ploeg

**Affiliations:** 1 Australian National Centre for Ocean Resources and Security (ANCORS), University of Wollongong, Squires Way, Wollongong, NSW 2500 Australia; 2 WorldFish, Jalan Batu Maung, Batu Maung, 11960 Bayan Lepas, Penang Malaysia; 3 College of Science & Engineering, James Cook University, Townsville, QLD 4811 Australia; 4 Department of Earth Sciences, Villavägen 16, 752 36 Uppsala, Sweden; 5 STINAPA Bonaire, Kralendijk, Netherlands

**Keywords:** Aquatic foods, Food security, Poverty reduction, Rural development, Small-scale fisheries, Sustainable livelihoods

## Abstract

Livelihood diversification has become an integral focus of policies and investments aiming to reduce poverty, vulnerability, and pressure on fishery resources in coastal communities around the globe. In this regard, coastal fisheries in the Pacific Islands have long been a sector where livelihood diversification has featured prominently. Yet, despite the widespread promotion and international investment in this strategy, the ability of externally funded livelihood diversification projects to facilitate improved resource management and rural development outcomes often remains inconsistent. We argue these inconsistencies can be attributed to a conceptual ambiguity stemming from a lack of attention and awareness to the complexity of livelihood diversification. There is still much to learn about the process of livelihood diversification, both in its theoretical conceptualizations and its practical applications. Herein, we utilize a common diversity framework to clarify some of this ambiguity by distinguishing three diversification pathways. These pathways are illustrated using an ideal–typical Pacific Island coastal household and supported by examples provided in the literature that detail livelihood diversification projects in the Pacific. Through this perspective, we seek a more nuanced understanding of what is meant within the policy and practice goal of livelihood diversification. Thereby enabling more targeted and deliberate planning for development investments that facilitates outcomes in support of sustainable livelihoods.

## Introduction

Within the last two decades, livelihood diversification has become an integral focus of policies and investments aiming to facilitate the dual objectives of poverty alleviation and fishery resource sustainability (Brugère et al. 2008; Haider et al. 2018). Institutionalization of livelihood diversification has also been prolific in the Pacific Islands where coastal fisheries are the backbone of local, national and regional economies, including food and nutrition security (King and Lambeth 2000; Gillett and Lightfoot 2001; O’Garra 2007). Approximately half of the people in the Pacific derive their primary or secondary source of income from coastal fisheries, and aquatic foods are the most widely consumed animal-sourced food by coastal people throughout the region, comprising between 50 and 90% of their animal-sourced protein (Bell et al. 2009; SPC 2015; Farmery et al. 2020).

Coastal fisheries provide nourishment for Pacific Islanders, but per capita coastal fisheries production is declining (Gillett 2016). To secure a sustainable supply of nutritious aquatic foods by supporting sustainable coastal fisheries, regional policies such as the Vava’u Declaration (FFA 2007), the “New Song for coastal fisheries” Noumea Strategy (SPC 2015), and the “Regional Roadmap for Sustainable Pacific Fisheries” (FFA & SPC 2015) emphasize livelihood diversification. This is an area of policy and practice where the Pacific Islands have seen significant investment into an array of projects to diversify coastal livelihoods through alternative or supplemental activities.

Yet, despite the widespread promotion and international investment in this strategy, the ability of livelihood diversification projects to reduce poverty and vulnerability while alleviating pressure on fishery resources often remains inconsistent (e.g., O’Garra 2007; Brugère et al. 2008; Gillett et al. 2008). For a long time scholars have argued that externally designed and funded projects are typically underpinned by misguided assumptions regarding the needs and desires of Pacific Island coastal communities (Johannes 1994; Foale 2001; Govan et al. 2019). The indicators of a successful livelihood from a Pacific Island perspective may not directly align to the indicators from a Western perspective, and these differences have important implications for ability of a livelihood project to achieve its intentions (Govan 2011). Experiences have demonstrated that livelihood projects in Pacific Island coastal fisheries that are not adapted to local contexts, including values and aspirations, are rarely continued after project funding ends (O’Garra 2007).

Linked to these misguided assumptions in practice is also conceptual ambiguity relating to what livelihood diversification is about and what it should look like. We argue this ambiguity stems from a failure to account for the complexity of livelihood diversification, which has contributed to the inconsistent fisheries and development outcomes observed thus far from diversification projects. For example, recent projects in the Pacific have supported fishers to diversify their livelihoods *within* the fishery sector by introducing new commercial species such as the trochus (*Rochia nilotica*) in Samoa (Purcell et al. 2021). Others have promoted alternative activities in sectors *outside* of capture fisheries, such as mariculture of ornamental species in Solomon Islands (WorldFish 2010) or ecotourism in Papua New Guinea (ACIAR 2018). While these examples are not mutually exclusive, they describe divergent diversification pathways, and each would contain a unique set of possible benefits and risks for different people in different contexts. It seems that a suite of very different approaches towards planning and intervention are subsumed under the concept of diversification.

In this perspective article, we unpack diversification into three distinct pathways to clarify the ambiguity around this concept as well as its empirical complexity. We do so utilizing the general framework for analyzing diversity in science, technology, and society introduced by Stirling (2007). This framework defines three properties of diversity: balance, variety, and disparity. We combine the properties of this general framework with the livelihood strategies defined in the sustainable livelihoods framework (Scoones 1998; Allison and Ellis 2001), a key international framework and analytical tool guiding livelihoods analysis and project planning. Combining these frameworks helps to clearly delineate the divergent pathways through which livelihood diversification is pursued and provides a fresh perspective on livelihood diversification as a concept employed in policy and planning. We distinguish these pathways using an ideal–typical Pacific Island coastal household and draw on examples provided in the literature that detail livelihood diversification projects in the Pacific to exemplify the pathways in practice. Subsequently, we describe how each diversification pathway can contribute to sustainable livelihoods, highlight some of the risks and caveats associated with them, and discuss the complexities that exist within and between them.

## Unpacking diversity

The emphasis on livelihood diversification is not unique to coastal fisheries or the Pacific; it has featured globally for decades now as a prominent strategy to improve resource management and rural development outcomes by reducing poverty, vulnerability and pressure on overexploited ecosystems (e.g., Nunan 2015; Roe et al. 2015; Haider et al. 2018). A livelihood consists of the portfolio of activities, material and non-material assets, and access to these that together support people’s lives (Ellis 2000). Through the process of livelihood diversification, individuals and households construct an increasingly diverse portfolio of livelihood activities and assets in order to survive, spread risk, and improve their standard of living. Although this process typically occurs endogenously as opportunity and capability allow, it has now also become the focus of many exogenous projects implemented by government agencies, non-governmental organizations, and international aid organizations.

By diversifying livelihood activities, individuals and households can reduce their exploitation levels of natural environments and increase their ability to adapt to changing economic, socio-political or environmental conditions (Torell et al. 2017). Flexibility in adaptation options and capacity to mobilize in order to pursue these options are intricately linked to sustainable livelihoods. Specifically, where assets and well-being can be maintained, vulnerability to external shocks and trends can be reduced, and livelihood activities do not overexploit natural resources and environments (Allison and Horemans 2006).

Since the emergence of the sustainable livelihoods framework in the 1990s (Chambers and Conway 1992; Scoones 1998), livelihood perspectives have become a central element of fisheries management and rural development research (Scoones 2009 offers a review of this evolution). The framework commonly defines three rural livelihood strategies that individuals and households employ to build sustainable livelihoods: intensification/extensification, diversification, and migration (see Scoones 1998). Parallelly, Stirling's (2007) common diversity framework has helped add texture and details to what diversity means across disciplines by breaking diversity into three interrelated properties: balance, variety, and disparity. Kotschy et al. (2015) applies these properties into a livelihood perspective by defining balance as the extent to which each (livelihood) option is currently practiced, variety as the number of options available, and disparity as the degree of difference between the options. Viewing the properties from Stirling's common diversity framework through the lens of the livelihood strategies defined in the sustainable livelihoods framework supports a deeper consideration of what diversification means and how management and policy can target livelihood diversification (Hanh and Boonstra 2018).

Table 1 juxtaposes Scoones’ sustainable livelihoods framework with Stirling's common diversity framework, and interprets their concepts in the contexts of livelihood diversification for Pacific Island fishery livelihoods. Considering this juxtaposition reveals some distinctions that need to be addressed when analyzing livelihood diversification in the context of Pacific Island fishery households. Specifically, we need to remember that Scoones developed the livelihoods framework based on research in East African pastoralist communities, which is why migration is a prominent feature (see Waller 1985). This agricultural context is also where the concept of extensification, or the cultivation of more land is derived from.

**Table 1:** Aligning the three rural livelihood strategies portrayed in the sustainable livelihoods framework and the three principles of diversity in the common diversity framework enables a interpretation for what diversification means in a completely different context: Pacific Island coastal fisheries

Scoones (1998) Sustainable livelihoods framework	Stirling (2007) Common diversity framework	Interpretation for Pacific Island coastal fishery livelihoods
Intensification/extensification	Balance	A livelihood enhancement to improve the production of an established activity within a household’s portfolio of activities
Diversification	Variety	The addition of a new livelihood activity to a household’s portfolio of activities
Migration	Disparity	The addition of (or substitution to) a new livelihood activity in a new economic sector to a household’s portfolio of activities

To analyze livelihood diversification in coastal fishery households we first make a distinction between diversification *within* fisheries versus diversification *outside* fisheries. With this distinction, the strategy of ‘migration’ depicted in the sustainable livelihoods framework is adjusted to focus on the pathway where fishers develop economic activities outside fisheries. Scoones’ original interpretation of geospatial migration in the context of Pacific coastal fisheries implies that people completely cease all fishery activities and migrate to urban centers in search of a modern lifestyle (e.g., Connell 2010). Additionally, we also focus solely on intensification (i.e., an efficiency improvement that allows fishers to increase their catch per unit effort) rather than devise a more fitting analogy for extensification in this context. What Scoones calls extensification may be analogous to the expansion of fishing grounds or fishing further from shore, but in the context of Pacific Island coastal fisheries, the capacity limitations of most fishers to access offshore fisheries, migratory nature of fishery resources, and traditional tenure systems would often inhibit such adaptations.

## Livelihood diversification pathways in a Pacific Island coastal fishery context

**Fig. 1:**
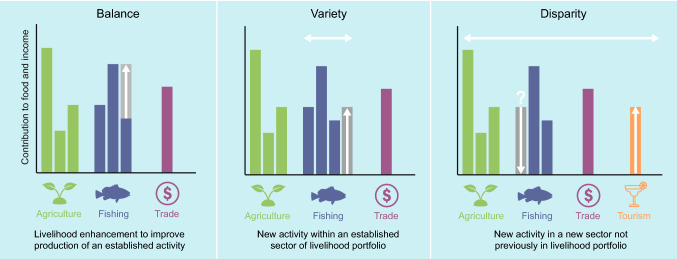
The three livelihood strategies adapted from the sustainable livelihoods approach (Scoones 1998) and their associated property of diversity (balance, variety, and disparity) from the common diversity framework (Stirling 2007). Increasing any or all of these three properties can increase the diversity of the overall livelihood portfolio and theoretically contribute to more resilient livelihoods

Based on the understanding that livelihoods in coastal households of the Pacific often engage in multiple activities such as fishing, farming, trading and tourism (e.g., Sulu et al. 2015), we construct a livelihood portfolio for an ideal–typical Pacific Island household to demonstrate the three diversification pathways (Fig. 1). To clarify, this ideal–typical household is not a description of a perfectly diversified household or a normative statement of how diversified these Pacific Island households would need to be. Ideal types emphasize and synthesize features of reality into an analytical construct (see Swedberg 2018). In our case this means that the ideal–typical household does not correspond directly to an existing household, but instead demonstrates what livelihood diversification can mean for fishing communities and households in the Pacific, and how livelihood diversification in this context can be understood. Our ideal type thus serves as a heuristic tool to reveal the complexity of livelihood diversification as theorized previously, and therefore should not be considered as a research finding.

The ideal–typical Pacific Island household in our example contains a portfolio of activities commonly employed by men and women throughout the region. In the Pacific, a household may cultivate three crops (e.g., taro, sweet potato, and coconut), employ three different fishing methods (e.g., spearfishing, gleaning shells, and fishing with a handline), and trade commodities such as canned tuna from a small store. Externally funded projects intending to diversify coastal livelihoods may promote activities that increase the balance, variety, or disparity of this household’s portfolio of livelihood activities.

### Balance

**Fig. 2:**
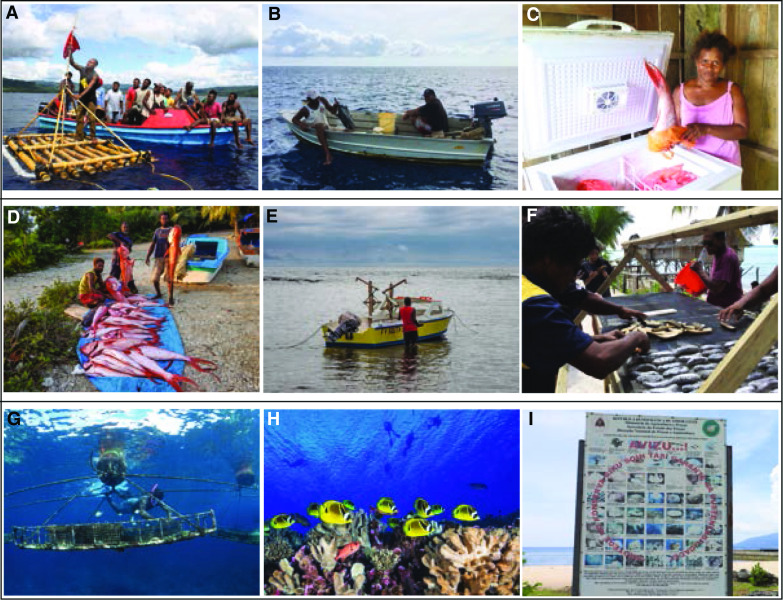
**A** Fishers deploying a fish aggregating device in Gwanatafu, Solomon Islands to catch pelagic species more efficiently. Photo by Hana Matsubara. **B** Fishers display their catch (Caranx melampygus) on an aluminum boat with an outboard motor in Tabuaeran, Kiribati. Photo by Jacob Eurich. **C** Woman holding a Lutjanidae fish tail that is being stored in a solar powered freezer in Surairo, Solomon Islands. Photo by Hampus Eriksson. **D** Fishers displaying their catch of deep-sea snapper in Aniwa Island, Vanuatu. Photo by Abel Sami. **E** Hard hull fishing boat with locally engineered fishing reels for deep-sea fishing in Tanna, Vanuatu. Photo by Dirk J. Steenbergen. **F** Processing sea cucumbers for export using mesh trays and solar heat in Tarawa, Kiribati. Photo by Aquaculture Unit, Ministry of Fisheries and Marine Resources Development (MFMRD). **G** Diver tends to a giant clam floating cage nursery off Nusatupe, Solomon Islands. Photo by Mike McCoy (**H**) Above the reef in Tahiti, French Polynesia tourists prepare to go scuba diving. Photo by Jayne Jenkins/Ocean Image Bank (**I**) A signboard displaying the protected species and habitats in Com, Timor-Leste. USAID’s Coral Triangle Support Partnership has helped communities such as Com, living inside the Nino Konis Santanta National Park, to brainstorm adaptation activities including diversifying income sources through development of small-scale tourism. Photo by Hampus Eriksson

Balance relates to the relative contribution of existing activities in the livelihood portfolio to food and/or income security. Enhancing the efficiency or altering the time, money, or effort invested in an activity already being employed can change the balance of the portfolio. For our ideal–typical household the balance of livelihood activities could change through obtaining an outboard motor that enables fishing activities (e.g., fishing with a handline) to be done more quickly, or a freezer that prolongs the shelf life and quality of fish for sale (Fig. 1A). Common examples from the Pacific Island literature describe technological innovations that increase fishing or post-harvesting efficiencies. For example, deploying fish aggregating devices (FADs) in areas where fishing for pelagic species already occurs such as in the Solomon Islands (Albert et al. 2014) and Timor-Leste (Tilley et al. 2019), or subsidizing modernized fishing vessels complete with outboard motors (Gillett and Moy 2006) can increase the yields of existing livelihood activities. Fisheries centers have often been constructed in the region to enhance the economic viability of existing coastal fishery activities (Gillett 2010). Alternatively, solar powered freezers have recently been piloted as a lighter touch approach to innovate with rural economic practices in the Solomon Islands (WorldFish 2019) (Fig. 2A–C).

Changing the balance of existing activities within livelihood portfolios can help enhance the accrual of capital assets, thereby assisting to better absorb external shocks and stresses. Building assets can also enable people to decouple their dependence on an activity once certain thresholds are met. Decoupling dependence is a particularly important consideration here, as putting too much investment into an activity can ultimately decrease the balance of the overall portfolio and increase vulnerabilities to external shocks and stresses through overdependence. To demonstrate, if our ideal–typical Pacific Island household had increased their accumulation of financial savings by obtaining an outboard motor that enabled a bigger catch per unit effort, they would be in a better position to cope with the adverse impacts of an extreme weather event or a global pandemic. But, if they remained specialized in that livelihood activity to the exclusion of others, they risk losing their only livelihood source if something were to happen to their boat.

When enhancing the efficiency of fish-based livelihood activities there is also risk of perpetuating overfishing and resource overexploitation, ultimately threatening livelihoods and degrading ecosystems. Gillett et al. (2008) describes this occurring in Fiji, where boats obtained from government subsidy were primarily used for spearfishing inshore, which led to increased depletion of fishery resources. Yet, often the poorest and most marginalized are unable to mobilize their limited assets to pursue different livelihood activities due to so-called 'poverty traps' (Carter and Barrett 2006). Once certain thresholds are met where decreasing dependence becomes possible, effort can be shifted into additional livelihood activities to restore balance of the overall portfolio. For our ideal–typical household, this could mean purchasing a cast net to catch Mugilidae fish species from the shore once they had enough financial savings to lessen dependence on their boat.

### Variety

Variety relates to how many activities comprise the livelihood portfolio. Adding a new livelihood activity increases the variety of activities in the livelihood portfolio. From a fisheries perspective, increasing the variety of activities embodies a shift towards a generalist strategy of fishing, where multiple species are targeted and multiple gear types are used. Members of our ideal–typical household may increase the variety of activities in their livelihood portfolio by employing an additional fishing activity such as purchasing a gill net to use from their canoe in coastal lagoons and reefs (Fig. 1B). Projects throughout the Pacific promote diversification within this pathway through the utilization of new fishing methods and techniques or targeting new species and functional groups. Introduced methods such as horizontal longline fishing (Beverly et al. 2003), or targeting deep-sea species such a snapper (Adams and Chapman 2004), or opportunistically collecting sea cucumbers to take advantage of changing government regulations and market conditions (Purdy et al. 2017) can all increase the variety of livelihood activities within the fisheries sector (Fig. 2D–F).

Increasing the variety of activities within livelihood portfolios may assist individuals and households adapt to external shocks and stresses by switching between livelihood activities as necessary (Allison and Ellis 2001). Globally, increasing catch diversity has been shown to help stabilize income and buoy the size and market value of catches (van Oostenbrugge et al. 2002; Kasperski and Holland 2013; Finkbeiner 2015), even amid long-term declines across multiple species groups (Robinson et al. 2020). Catch diversity can also enable a more balanced allocation of fishing effort to alleviate pressure on highly commoditized coastal fishery resources (Gillett et al. 2008; Finkbeiner 2015).

Ideally, the variety of activities are distributed across multiple types of ecosystems. Utilizing a broad range of ecosystems can help prevent overexploitation of specific species or habitats, thus minimizing impact on ecosystem services and helping to safeguard desirable ecosystem states that support livelihoods and food security. It may also spread risk in case of adverse regime shifts in ecosystem states from larger scale processes such as climate change (see Folke et al. 2004 for a review of regime shifts). For example, if the fishing activities conducted by our ideal–typical household all took place in the shallow lagoon, they would all put fishing pressure on the same habitat, and they would all still be vulnerable to a coral bleaching event. However, if their new gill net allowed them to fish on the outer reef where the water is deeper and cooler, they would be able to adapt to this bleaching event in the lagoon by shifting their effort to different environments such as the outer reef.

Conversely, there are also several risks associated with adding more livelihood activities into a household portfolio. Doing so could result in an added labor burden, particularly for women who still have disproportionate domestic responsibilities compared to men (e.g., Lawless et al. 2019). Or it could require access to ecosystems outside of traditionally tenured areas that may create new conflicts (e.g., Albert et al. 2014). If unmitigated these factors can undermine the positive attributes of increasing the variety of livelihood activities in portfolios, for example by deteriorating local social capital. First considering gender and social norms such as the differences in women’s and men’s divisions of labor (Stacey et al. 2019), or local tenure rights and taboos (Foale et al. 2011) can help minimize these risks. Simply, livelihood solutions proposed through diversification projects need to fit and function within local social and ecological environments. Particularly in the Pacific, engaging with customary knowledge institutions may assist externally funded projects ensure that proposed solutions are aligned to the sustainable use of local environments.

### Disparity

Disparity relates to how different from each other are the activities in the portfolio. Adding another livelihood activity in a new and different economic sector increases the disparity of activities in the livelihood portfolio. This new livelihood activity may or may not substitute for a previously employed livelihood activity. Embedded within this pathway is the concept of alternative livelihood activities, which are broadly used as an approach to achieve biodiversity conservation objectives by foregoing ecologically harmful activities for activities that have less of an impact on natural resources (Roe et al. 2015).

A prevalent example in a Pacific Island coastal fishery context is when fish-based livelihood activities are substituted for alternative eco-tourism activities in parallel with the implementation of a marine reserve established under customary tenure arrangements (Aswani and Weiant 2003; Brunnschweiler 2010). For example, through the assistance of an externally funded project our ideal–typical household may decide to participate in the ecotourism industry by building a guesthouse. As a result of this, perhaps they also decide to stop spearfishing in the lagoon that has now been established as a marine reserve (Fig. 1C).

There is a plethora of externally funded projects in the Pacific that detail diversification through a transformation into new sectors such as aquaculture or tourism. Adding new activities such as seaweed and giant clam farming (O’Garra 2007), or substituting a resource extractive activity in favor of eco-tourism (Wood et al. 2013; Addinsall et al. 2017) can increase the disparity of activities in livelihood portfolios (Fig. 2G–I). At the extreme end of this pathway is outward migration, where people move to domestic and international urban centers in pursuit of new opportunities. Frequently, those that migrate help increase the disparity of livelihood portfolios for family that remain at home through remittances (Connell 2010). In some contemporary rural and semi-rural settings, remittances make up a significant contribution to household and village economies (Sulu et al. 2015).

In its most simplified form, transforming livelihood activities into new economic sectors has the potential to improve income, reduce vulnerabilities and reduce pressure on overexploited natural resources by minimizing dependence on them; and there are numerous instances where externally funded projects have facilitated these outcomes globally. Increasing the diversity of livelihood activities across economic sectors has helped decrease poverty and vulnerability by providing access to material assets like income (Pant et al. 2014; Torell et al. 2017) and non-material assets such as human and social capital (Fröcklin et al. 2018). It has also reduced pressure on overexploited resources by shifting effort away from the fishery sector (Torell et al. 2017). However, the degree to which individuals or households can reduce their dependencies on coastal fishery resources through livelihood diversification depends on their desires as well as the opportunities and capacities they have to diversify. For example, land ownership can be a pre-requisite for agricultural food and cash crop production (e.g., Sulu et al. 2015).

Externally funded projects often do not consider or differentiate for the desires, opportunities, or capacities that various households have (O’Garra 2007). Projects promoting alternative activities in new economic sectors are often built on flawed assumptions about individuals substituting environmentally damaging livelihood activities for sustainable ones, homogenous communities composed of households with common characteristics, and livelihood alternatives scaling from individuals to entire communities and populations (Wright et al. 2015). Diversification through this pathway usually requires overcoming steep barriers of entry (e.g., land access) and financial investments that inhibit participation. For our ideal–typical household, their ability to participate in the ecotourism sector by building a guesthouse is contingent upon their control over and access to several types of assets, including natural and financial capital. Even if possible, evidence from southeast Asia shows that leaving the fishery for an alternative livelihood activity may not be a desirable option due to the cultural significance of fishing and non-material benefits gained from it (Pollnac et al. 2001). These examples indicate that it is crucial to match project ambitions with the ecological and political economies in which people are situated, and enable or limit diversification from occurring the way the project intends.

Yet, the future of small-scale fisheries in the Pacific Islands, including the livelihoods that depend on them, may hinge upon the development of alternative subsistence and income sources (Kronen et al. 2010). Efforts to diversify into alternative livelihood activities need to be tailored to local contexts to be effective, and build on existing strengths and institutions (Govan 2011). In this regard, diagnostic tools that help guide conversations about potential new livelihood activities can facilitate the identification of locally tailored ideas as well as potential problems and risks of these proposed activities (e.g., Govan et al. 2019). Diagnosis of how diversification in terms of balance, variety, and disparity is influenced by the desires, opportunities, and capacities that various households hold can help ensure a positive trajectory of transformations into new livelihood sectors and substitutions into alternative livelihood activities (Diedrich and Aswani 2016).

### The complexity of livelihood diversification

The inconsistent poverty reduction and resource sustainability outcomes from externally funded projects is likely to come from the inherent complexity that characterizes livelihood diversification. Balance, variety, and disparity are not separate qualities of livelihoods but are tightly interdependent. For example, when adding another livelihood activity, the balance of the entire portfolio is naturally impacted as effort across all activities is reallocated to incorporate the new activity. Alternatively, substituting several previously developed livelihood activities for a singular activity in a new and different economic sector may increase disparity but reduce the overall variety of activities in the portfolio. Individuals and households may also move in and out of different pathways as desires, opportunities, and capacities allow them to do so. Illustrative of this is how fishers sometimes switch between specialist (balance) and generalist (variety) fishing strategies during favorable conditions (Smith and McKelvey 1986; Finkbeiner 2015).

The potential to reduce poverty, vulnerability, and pressure on overexploited ecosystems within a given diversification pathway is also contingent. Although increasing the variety or disparity of livelihood activities can increase the utilization of diverse resources, if these activities are still reliant on the same broad ecosystem services in a limited spatial range, they are still vulnerable to the same large-scale threats such as climate change (Ellis 2000; Goulden et al. 2013). Simply, transforming into aquaculture-based livelihoods can increase the disparity of the livelihood portfolio and help reduce pressure on coastal fishery resources by shifting effort out of the capture fishery sector. However, it may not minimize exposure to increasingly more frequent extreme weather events as well as transforming into a non-natural resource-dependent sector could.

Furthermore, depending on the point of departure a given pathway within one household may represent an entirely different pathway in a different household. To demonstrate, if members from our ideal–typical household already fish for pelagic fish species from their canoe with a handline and then a FAD is deployed, this embodies a livelihood enhancement that increases the efficiency of a pre-existing activity (i.e., balance). For a different household that only fishes in the lagoon with a spear, obtaining the means to utilize the FAD for fishing represents a new activity (i.e., variety). These important distinctions can enable more targeted and deliberate planning for policies and investments that facilitate outcomes in support of sustainable livelihoods based on diverse needs and opportunities.

## Conclusion

Previous literature has pointed to misguided assumptions and maladapted solutions as cause for the inconsistent fishery and development outcomes from externally funded diversification projects in the Pacific. Our perspective is that at least in part, these inconsistencies can also be attributed to conceptual ambiguity stemming from a lack of attention and awareness to the complexity of livelihood diversification. Conventional understandings and definitions of livelihood diversification typically fail to capture this complexity due to pre-conceived ideas about material assistance and “livelihood projects”. In practice, diversification can mean different things for different people depending on their living circumstances, and often involves making hard choices between economic development and resource conservation.

Aligning Stirling's (2007) three properties of diversity with Scoones' (1998) rural livelihood strategies helps to highlight the complexity within livelihood diversification and unpack some of the ambiguity around diversification as a concept. The relevance and empirical manifestation of the diversification pathways we identified from this effort were illustrated with examples provided in the literature of livelihood projects and studies in Pacific Island coastal fisheries. Making analytical distinctions between the balance, variety, and disparity of livelihood diversification can help to better understand the contingency of the effects it has on poverty, vulnerability, and ecosystems. It therefore remains a continuous question and significant research frontier to know how this causality plays out in specific social and ecological contexts.

Based on these conclusions we suggest that future projects seeking to facilitate development or conservation through livelihood diversification would do well to articulate more deliberately the goals to which livelihood diversification should contribute. A more deliberate approach could assist external partners to shift towards extension-based service and information provision based on the circumstances in target beneficiary communities. It can also help recognize the risks of exposing new vulnerabilities through maladapted diversification processes that lead to unintended trade-offs or consequences within these communities. Thus, leveraging the potential of diversification to realize positive-sum outcomes where both development and conservation of fisheries resources can be achieved, or instead when livelihood diversification cannot achieve these goals at the same time. Given the emphasis on livelihood diversification as a policy goal in both Pacific Island coastal communities (e.g., SPC 2015, p. 10), and for global small-scale fisheries (e.g., FAO 2015: Sect. 6.8), this advice for external agencies and partners has significant practical application and meaning to help achieve the unique development challenges that coastal communities in the Pacific face.
